# Editorial: Extracellular traps in cancer immunity and immunotherapy

**DOI:** 10.3389/fimmu.2023.1292819

**Published:** 2023-09-26

**Authors:** Michal A. Rahat, Maria Rosaria Galdiero

**Affiliations:** ^1^ Immunotherapy Laboratory, Carmel Medical Center, and the Rappaport Faculty of Medicine, Technion-Israel Institute of Technology, Haifa, Israel; ^2^ Department of Translational Medical Sciences (DiSMeT), Center for Basic and Clinical Immunology Research (CISI), World Allergy Organization Center of Excellence, University of Naples, Federico II, Naples, Italy

**Keywords:** neutrophil extracellular traps (NETs), macrophage extracellular traps (METs), tumor immunity, cancer immunotherapy, citrullinated histone 3, neutrophil elastase (NE), myeloperoxidase (MPO), tumor microenvironment (TME)

Neutrophils and macrophages are the main cell types known to cast extracellular traps (ET), composed of DNA and histones (mostly in their citrullinated form), and further decorated by different proteins (1). When neutrophils undergo a special kind of cell death known as NETosis, they cast the neutrophil extracellular traps (NETs) that include proteins such as neutrophil elastase (NE) and myeloperoxidase (MPO) (2). Similarly, macrophages die by METosis, and cast macrophage extracellular traps (METs), that present only minor differences compared to NETs, such as shorter chromatin fragments and faster formation (3, 4). Other cell types such as eosinophils and lymphocytes can also cast ETs, although their significance is not sufficiently studied.

NETs and METs were first discovered in the context of infection, as they are able to trap bacteria and limit their spread (1), but they are also involved in many inflammatory and autoimmune diseases, as well as in cancer (5).

Two review papers examine the cross talk between tumor cells and NETs. Zhao and Jin review the role of NETs in promoting tumor progression in different tumor models and human patients. NET-associated HMGB1 or NE can bind to TLR9 or TLR4, respectively. This triggers tumor cells to enhance their proliferation, increase mitochondrial biogenesis, and promote the release of cytokines such as IL-6 and IL-8, which in turn, prime neutrophils to cast more NETs. Chronic inflammation increases NETs formation, which remodels the extracellular matrix (ECM) due to the presence of proteases such as MMP-9 or proteinase 3 (PR3). The degraded ECM proteins, specifically laminin, promote the exit of tumor cells from dormancy. NETs also affect resistance to therapy. After chemotherapy or radiotherapy, tumor cells that die release DAMPs that increase NETs formation. The mesh of chromatin protects tumor cells from NK cell or CD8+ T cell cytotoxicity, likely through the NET-associated PD-L1.

Fang et al. highlight the crosstalk between NETs and different immune cells in the tumor immune microenvironment (TIME). Tumor cells promote the formation of PD-L1-embedded NETs by secreting G-CSF and IL-8. These NETs lead to CD8+ T cell exhaustion and dysfunction, manifested by the increased levels of PD-1, Tim3 and Lag3, and the decreased production of IL-2, IFNγ and TNFα. Additionally, NETs protect tumor cells by providing a physical barrier and prevent the contact between tumor and stroma cells. NETs co-localized with Tregs, suggesting that they promote their differentiation from naïve CD4+ T cells or alternatively, that Tregs promote NETs formation. Thus, NETs promote the immunosuppressive functions of Tregs, NK cells and CD8+ T cells. Macrophages that are dominant in the TIME, can either promote NETs formation or enhance their clearance through secretion of DNAse and phagocytosis, depending on the mode of activation of the macrophage and on the surrounding TME. Therefore, the overall activity of NETs, that either improves anti-cancer response or protects tumor cells, probably depends on the complex interactions between the tumor cells, the NETs and the TIME.

Along those lines, Kaltenmeier et al. provide *in vivo* evidence for the importance of PD-L1 expression in NETs on the exhaustion of T cells. NETs containing PD-L1 that were injected into tumors or enriched by liver ischemia/reperfusion in a liver-metastatic mouse model, resulted in enhance expression of inhibitory receptors on infiltrating CD4+ and CD8+ T cells and a metabolic exhaustion phenotype. This was dependent on the expression of PD-L1, as this phenotype did not occur in NETs derived from PD-L1 KO mice. Furthermore, T cell exhaustion was reversed by disrupting NETs using DNAse or anti-PD-L1 injections, resulting in smaller tumors. Neutrophils from patients with colorectal liver metastases after resection that were primed to form NETs, induced exhaustion of CD4+ and CD8+ human T cells. Thus, NETs induce T cell exhaustion to promote tumor growth, and targeting NETs by anti-PD-L1 reduces tumor growth.

The next two papers describe NETs biomarkers. Tomás-Pérez et al. characterize biomarkers of NETosis in high-grade serous ovarian cancer (HGSOC). They propose that NETosis promotes cancer, as formation of NETs allows the capturing of metastatic cells in the peritoneal fluid and facilitates their colonization on the omentum surface. Five specific biomarkers (cell-free DNA (cfDNA), DNA-nucleosomes complexes, calprotectin, MPO and DNA-citH3 complexes) were chosen, and their levels were determined in samples from peripheral blood or peritoneal fluids obtained from HGSOC and control women. The increased levels of these biomarkers, especially in the peritoneal fluid in the cancer patients suggested a possible contribution of NETosis in HGSOC. Neoadjuvant treatment significantly reduced the levels of these NETosis biomarkers mostly in the peripheral blood and not in the peritoneal fluids, indicating that the impact of treatment on the process of NETosis.

Modestino et al. measured serum concentrations of NET biomarkers and neutrophil-related mediators in patients with differentiated thyroid cancer (DTC), dedifferentiated thyroid cancer (De-DTC), multinodular goiter (MNG) and healthy controls (HCs). They found that **s**erum levels of all four NET biomarkers were increased in DeDTC patients compared to HCs. CitH3 serum levels were selectively increased in both DeDTC and DTC patients compared to HCs and MNG patients. MPO-DNA complexes and nucleosomes were selectively increased only in DeDTC patients compared to HCs and MNG patients. Moreover, MPO-DNA complexes were selectively increased in DeDTC patients compared to DTC patients also. Circulating levels of neutrophil-related mediators were increased in DTC and DeDTC patients compared to HCs. These data suggest that MPO-DNA complexes, nucleosomes and, to some extent, CitH3 levels correlate with malignancy and severity of progressive TC.

Cheng et al. constructed a four-gene signature prognostic model based on the enterocyte-related differentially expressed genes (ERDEGs) in patients with colon adenocarcinoma. The risk score derived from this model was considered as an independent variable factor to predict overall survival. The patients were divided into high- and low-risk groups based on the median risk score value. The IFN-γ scores were low, and T cell dysfunction scores were high in the patients in the high-risk group. A positive correlation between the risk score and macrophages was observed, but a negative correlation was observed between plasma cells, dendritic cells, resting mast cells, resting memory T cells, and risk score. These results suggest that the four-gene signature prognostic risk model could accurately predict the status immune microenvironment of patients with colon adenocarcinoma. Infiltrating neutrophils encounter the TME that include Peptidases, ions and metabolic signaling mediators, which are partly regulated by the four genes identified in the signature. Theses mediators help regulate NETosis and NETs formation. Therefore, although not directly stated, changes in the four-gene signature might also suggest changes in NETs formation.

Collectively, this series of articles highlight the importance of extracellular traps from neutrophils (NETs) and macrophages (METs) on cancer progression, suggests new mechanisms for tumor advancement that involves NETs, and places those traps as new therapeutic targets to inhibit tumor progression and metastasis.

## Author contributions

MR: Writing – original draft, Writing – review & editing. MG: Writing – original draft, Writing – review & editing.
